# Supplementary material for: Predictors of death among severe COVID-19 patients admitted in Hawassa City, Sidama, Southern Ethiopia: Unmatched case-control study
Source: PLoS One. 2023 Mar 1;18(3):e0282478. doi: 10.1371/journal.pone.0282478 (PMC9977030; doi:10.1371/journal.pone.0282478)
Supplement: Attachment — Submitted filename: Response to Reviewers.docx [file pone.0282478.s001.docx]

**PLOS ONE	Date: November 2022**

**Author's point to point response to editor and reviewers comments**

**Title**: Predictors of death among severe COVID-19 patients admitted in Hawassa City, Sidama, Southern Ethiopia: Unmatched case-control study

**Manuscript ID: PONE-D-22-21411**

**Subject: Submitting a revised version of the manuscript**

**Dear editor/reviewers.**

We would like to thank you for your constructive and valuable comments and suggestions that would improve the substance and content of the manuscript. We have addressed all comments and suggestions one by one and hence revised the manuscript thoroughly. Our point-by-point responses for each comment and suggestion are described below.

Thank you for considering our manuscript again

**Response to editor’s comment**

**Comment 1:** Please ensure that your manuscript meets PLOS ONE's style requirements, including those for file naming.

**Author’s response: Thank you editor, for the comments.** We have revised our manuscript based on journals style.

**Comment 3:** We note that you have indicated that data from this study are available upon request. PLOS only allows data to be available upon request if there are legal or ethical restrictions on sharing data publicly.

**Author’s response: Thank you editor, for the comments.** We have revised our cover letter and the data are available upon responsible request and we have mentioned the reason for restriction in the manuscript.

**Response to Reviewers comments**

**Reviewer #1**

**Comment 1:** The authors should follow the same pattern to represent the numbers. In some places it is written like 5,998,863 whereas in some other places simply 55 000 is written.

**Authors' response**. Thank you reviewer for the comment. The manuscript has been extensively edited (Line 64, 65, Page 4).

**Comment 2:** Lines 68-69 include both the references in the same [].

**Authors’ response: Thank you reviewer, for the comments**. We have corrected based on your comment in the revised manuscript (Line 84, 85, page 5).

**Comment 3**: There are lots of formatting issues and grammatical mistakes in the manuscript. For instance in line 71 no space between (chronic kidney disease[25])

**Authors’ response**: **thank** **you** **reviewer**, **for** **your** **comment**. We have revised and wrote it concisely in the revised manuscript (Line 86, page 5).

**Comment 4:** The literature survey is not adequately done. A similar perspective (https://doi.org/10.3389/fcimb.2021.674277) was published recently and the authors should compare their results with that of the similar work.

**Authors’ response**: **Thank you reviewer, for your concern**. We tried to dig out more literatures and added them in the revised manuscript. We incorporated the literature given in the comment section. (Line 83, line 324, line 350)

**Comment 5**: Lines 80-81. Please correct the statement “But, majorities of studies on predictor of death and disease severity have been conducted with the context of Asian and American countries”. Please, see this article where data from around the world was used (https://doi.org/10.3389/fcimb.2021.674).

**Authors’ response: Thank you reviewer, for your concern and information;** we have re-wrote the statement and included it in the revised manuscript including given literature. (Line 95-96, Page 5).

**Comment 6**: Line 93, 121 spellings of severe

**Authors’ response**: **thank** **you** **reviewer**, **for** **your** **comment**. We corrected the spelling error in the revised manuscript. (Line 93, and Line 135).

**Comment 7:** Please check the spellings of severe in full manuscript.

**Authors’ response**: **thank** **you** **reviewer**, **for** **your** **comment**. The manuscript has been extensively edited and corrected for grammatical and spelling error by all the authors and native English speaker who works in our University.

**Comment 8**: Line 255. Gap between 223(59.9%)

**Authors’ response**: **thank** **you** **reviewer**, **for** **your** **comment**. The edition error was corrected in the revised manuscript. (Page 14 line 267).

**Comment 9:** In some parts of the manuscript, COVID-19 is written whereas in some parts covid-19 is there. Please follow the same notion throughout the manuscript.

**Authors’ response**: **thank** **you** **reviewer**, **for** **your** **comment;** the capitalization error have been corrected in the revised manuscript. (Line 300, 362, 542)
