# Supplementary material for: Predictors of death among severe COVID-19 patients admitted in Hawassa City, Sidama, Southern Ethiopia: Unmatched case-control study
Source: PLoS One. 2023 Mar 1;18(3):e0282478. doi: 10.1371/journal.pone.0282478 (PMC9977030; doi:10.1371/journal.pone.0282478)
Supplement: Attachment — Submitted filename: Response to Reviewers.docx [file pone.0282478.s002.docx]

**PLOS ONE	Date: January 2023**

**Author's point to point response to editor and reviewers comments**

**Title**: Predictors of death among severe COVID-19 patients admitted in Hawassa City, Sidama, Southern Ethiopia: Unmatched case-control study

**Manuscript ID: PONE-D-22-21411R1**

**Subject: Submitting a revised version of the manuscript**

**Dear editor/reviewers.**

We would like to thank you for your constructive and valuable comments and suggestions that would improve the substance and content of the manuscript. We have addressed all comments and suggestions one by one and hence revised the manuscript thoroughly. Our point-by-point responses for each comment and suggestion are described below.

Thank you for considering our manuscript again

**Response to editor’s comment**

**Comment 1:** Of particular note, please address the concerns of the reviewer regarding the literature search in the Introduction, the advancement in knowledge from the study.

**Author’s response: Thank you editor, for the comments.** We have addressed the comments of the reviewer and incorporated all the necessary information from literatures and the important findings of this study. This can be traced from the revised manuscript.

**Response to Reviewers comments**

**Reviewer #1**

**Comment 1:** This is a case control study. The data for control was obtained from May 2021 to June 2021 whereas severe COVID-19 deaths were included from a period of May 2020 to May 2021. There is the disparity of the timelines and the authors should discuss the repercussions of taking the different timelines for the analysis.

**Authors' response**. **Thank you reviewer for the comment**. There is no time disparity between the sampling of cases and controls. This case- control study was done using similar timeline from May 2020 to May 2021. The period from May 2021 to June 2021 is the period of data collection for both case and control. The manuscript has been extensively edited.

**Comment 2:** In continuation to the above point the virus has changed a lot during the case-control timelines. The authors should discuss how the mutations in the virus can lead to variations in the results.

**Authors’ response: Thank you reviewer, for the comments**. This study takes secondary data (patient chart) retrospectively which did not consider the emergency of new variant across the study period. Therefore the finding did not indicate the effect of new variant virus on the disease outcome. This is mentioned as the limitation of the study which shall be investigated more (Line; 429-430).

**Comment 3**: The literature search in the introduction section is not adequate. The authors should discuss similar studies including PMID = 34760713.

**Authors’ response**: **Thank you reviewer, for your concern**. We tried to dig out more literatures and added them in the revised manuscript. We incorporated the literature given in the comment section. Among the literatures added; (Line; 57-58, Line; 66-76, Line; 106-108, Line; 119-120).

**Comment 4:**  It is unclear whether age-matched controls were used in the study.

**Authors’ response**: **thank** **you** **reviewer**, **for** **your** **comment**. The age of the patient is not matched and it is used as one variable found to be independent predictor of death. The extensive analysis and work up can be seen from the manuscript.

**Comment 5**:  It is not clear what knowledge is being added by this study. The conclusions made in the study have already been published by several groups. The authors must discuss any unique points that this study caters to.

**Authors’ response: Thank you reviewer, for your concern and information**; the major findings and the unique for this study has been included in the revised manuscript. The study is the only study done in Southern Ethiopia outside Addis Ababa. The sample size is larger and the sample of cases was taken to be all the deaths in the study period which gives credibility and power. This study found more number of predictors than other studies done in Ethiopia.

**Comment 7:** The data was analyzed using SPSS 25. The authors should provide the codes also so that the study can be reproduced.

**Authors’ response**: **thank** **you** **reviewer**, **for** **your** **comment**. The data availability has been mentioned in the manuscript. The nature of data which is sensitive and not good to share is mentioned. But, the data can be obtained up on responsible request.
